# Atypical Chest X-Ray Calcification in an Idiopathic Constrictive Pericarditis Case

**DOI:** 10.1155/2013/609610

**Published:** 2013-07-10

**Authors:** Uğur Coşkun, İsmail Polat Canbolat, Ümit Yaşar Sinan, Cem Bostan, Kadriye Kılıçkesmez, Ahmet Yıldız, Murat Başkurt, Fatma Nihan Turhan Çağlar, Alican Hatemi, Cenk Eray Yıldız, Sadettin Cöhcen, Aziz Tevfik Gürmen, Mehmet Serdar Küçükoğlu

**Affiliations:** ^1^Istanbul University, Institute of Cardiology, Cardiology Department, Haseki Street, Fatih, 34096 Istanbul, Turkey; ^2^Istanbul University, Institute of Cardiology, Cardiovascular Surgery Department, Haseki Street, Fatih, 34096 Istanbul, Turkey

## Abstract

Constrictive pericarditis is an uncommon cause of heart failure. It is a clinical entity caused by thickening, fibrosis, and/or calcification of the pericardium. We present a 50-year-old female patient who was admitted to our institution with a 6-month history of progressive dyspnea on exertion, abdominal swelling, and lower extremity edema. Her chest X-ray revealed an oblique linear calcification in the cardiac silhouette. Transthoracic echocardiography revealed biatrial enlargement. Left ventricular size and systolic function were normal. Cardiac computed tomography revealed the pericardial thickening (>5 mm) and heavy calcification in left atrioventricular groove. Simultaneous right and left heart catheterization showed elevation and equalization of right-sided and left-sided diastolic filling pressures, with characteristic dip, and plateau. Pericardiectomy was performed which revealed a thick, fibrous, calcified, and densely adherent pericardium constricting the heart. The postoperative period was uneventful and was in NYHA functional class I after 3 months.

## 1. Introduction

Constrictive pericarditis (CP) is uncommon cause of heart failure. It is a clinical entity caused by thickening, fibrosis, and/or calcification of the pericardium. This entity often leads to impairment of diastolic filling, resulting predominantly in symptoms of right heart failure [1]. Currently, idiopathic or viral pericarditis is the predominant cause in the industrialized countries, followed by cardiac surgery and mediastinal irradiation, which are as well the major and increasing causes of CP in the industrialized countries [2–4]. Tuberculosis is still a common cause of CP in developing and underdeveloped countries, as well as in the immunosuppressed patients [5]. Modern series from Saudi Arabia, Mexico, Turkey, and India document tuberculosis in 38% to 83% of all cases of CP. Pericardial disease rarely presents as the initial manifestation of tuberculosis [6–9]. Although pericardial calcification on chest X-ray suggests constriction, it is not diagnostic but may lead to more detailed investigations. A pericardial thickness less than 2 mm is normal and greater than 6 mm in size is specific for constriction [10]. Cardiac CT and MRI can detect pericardial thickening and calcification with high accuracy [11]. Echocardiography and new Doppler techniques are very useful for differential diagnosis between CP and restrictive cardiomyopathy [12, 13]. The gold standard for diagnosis is cardiac catheterization with analysis of intracavitary pressure curves, which are high in end diastole and equal in all chambers. The diastolic profile in both ventricles presents the typical dip-and-plateau pattern and the difference between the diastolic pressures of both ventricles should not exceed 3–5 mmHg. Early diagnosis remains challenging and may require multimodal cardiac imaging. Confirming the diagnosis of constrictive pericarditis is crucial, since surgical intervention may be the only method for alleviating the symptoms.

## 2. Case Report

A 50-year-old female patient was admitted to our institution with 6-month history of progressive dyspnea on exertion, abdominal swelling, and lower extremity edema. Three months prior to her visit to our institution, the patient's treatment was initiated in another medical service, with suspicion of liver disease, and she was given furosemide. However, her condition progressively worsened with extreme swelling of the lower limbs and abdomen, significant limitations on effort, and difficulty to walk and to lie down in the supine position. She had no past medical history.

 On physical examination at admission to the cardiology department, the patient was hemodynamically stable (blood pressure 100/60 mmHg and pulse 100 beat per minute irregular), tachypneic (24 cpm), and not cyanotic (92% oxygen saturation in room air) but pale. Her pulse was strong, irregular and symmetrical. Cardiac and pulmonary auscultation revealed a low intensity arrhythmia, normal S1 and S2 sounds, and diminished breath sounds in both lung bases, accompanied by crackles. There was marked jugular venous distension up to the mandibular angle, painless hepatomegaly (liver palpable 5 cm below the right costal margin on the midclavicular line), positive hepatojugular reflux, and lower limb edema up to the knee (+++/++++). 

**Figure 1:**
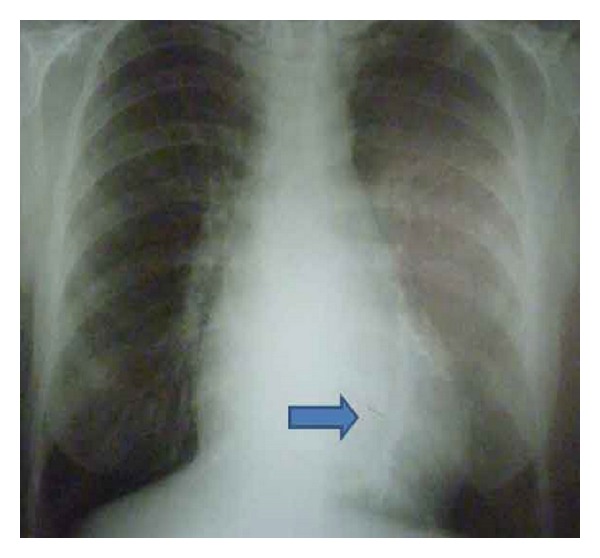
Oblique linear calcification (thick arrow) in chest X-ray.

**Figure 2:**
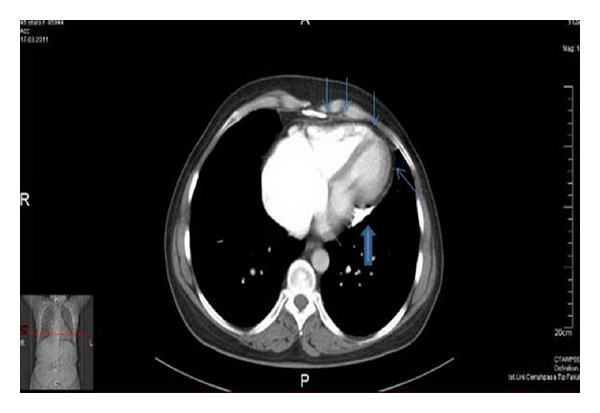
Pericardial thickening (thin arrows) and heavy calcification of left atrioventricular groove (thick arrow) in cardiac CT.

**Figure 3:**
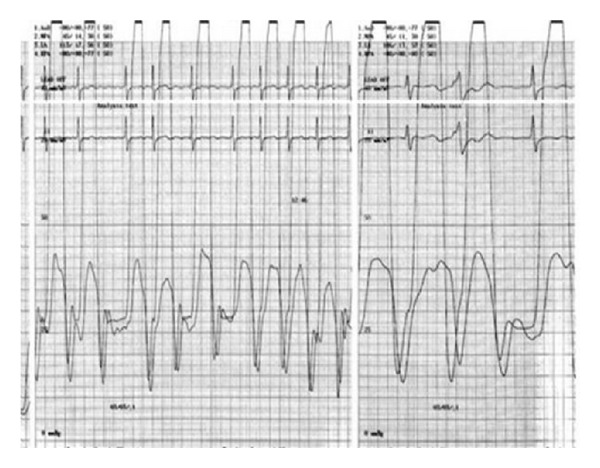
Equalization of right sided and left-sided diastolic filling pressures, with characteristic dip, and plateau in right-left cardiac catheterization.

**Figure 4:**
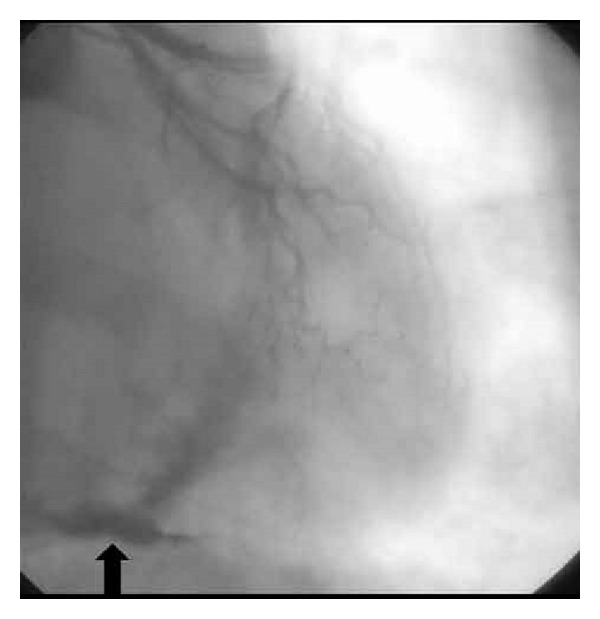
In coronary angiography during fluoroscopy linear calcifications (thick arrow) around the heart.

**Figure 5:**
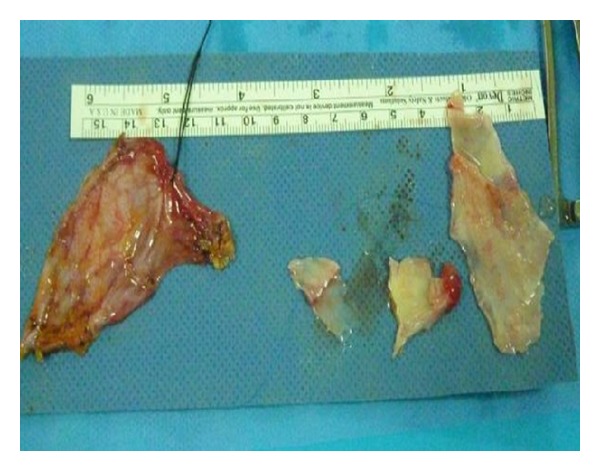
Macroscopic thick pericardial pieces.

 Biochemical laboratory tests were in normal limits. Cardiac enzymes were normal, and brain natriuretic peptide (BNP) was slightly above normal, at 140 pg/mL (0–125 pg/mL). Chest X-ray showed an oblique linear calcification in the cardiac silhouette (Figure 1). The electrocardiography revealed atrial fibrillation with diffuse nonspecific T-wave inversions. Transthoracic echocardiography revealed biatrial enlargement. Left ventricular size and systolic function were normal. Doppler transmitral inflow velocities revealed a restrictive physiology, however, tissue Doppler velocities of the mitral annulus were normal (10 cm/sec). Inferior vena cava was dilated (25 mm), with reduced respiratory variation (<50%). Cardiac computed tomography (CT) revealed the pericardial thickening (>5 mm) and heavy calcification in left atrioventricular groove (Figure 2). Simultaneous right and left heart catheterization showed elevation and equalization of right sided and left-sided diastolic filling pressures, with characteristic dip and plateau (Figure 3). Coronary angiography was normal. During fluoroscopy, linear calcifications around the heart were seen, similar to the chest X-ray (Figure 4). All of the findings were consistent with constrictive pericarditis. Pericardiectomy was performed, which revealed a thick, fibrous, calcified, and densely adherent pericardium constricting the heart (Figure 5). Pathology revealed a pericardium thickness of 0.5 cm, diffused fibrous tissue cells, nonspecific inflammatory cells, acellular hyalinization, and several focal dystrophic calcifications. The postoperative period was uneventful, and the patient was discharged from our hospital one month after admission with significant improvement, and she was in NYHA functional class I after 3 months. 

## 3. Discussion

The diagnosis of constrictive pericarditis should be seriously considered in patients with signs of chronic right side heart failure with exertional dyspnea, lower limb edema, mild liver congestion, and chest X-ray calcification in the presence of normal ventricular contraction. Other cardiac diseases, in particular right atrial myxoma, tricuspid valve dysfunction, and restrictive cardiomyopathy, must be ruled out [14]. Our patient had right side heart failure symptoms and an atypical chest X-ray calcification. This led us to consider additional investigations. Therefore, we have performed cardiac CT and cardiac catheterization. The investigations allowed us to confirm the diagnosis of constrictive pericarditis. Our patient had no previous medical history of pericarditis, tuberculosis, open-heart surgery, chest trauma, or radiotherapy. Therefore, we ended up with the definitive diagnosis of idiopathic constrictive pericarditis. 

Ling et al. reported roentgenological localization of pericardial calcification in 36 out of 135 patients with constrictive pericarditis, who underwent pericardiectomy. They mostly found pericardial calcification on the inferior-diaphragmatic border of the heart, rather than on the right ventricular area and the end of left atrioventricular groove [15]. We consider that our patient's chest X-ray findings were different from those usually presented in constrictive pericarditis. 

In conclusion, in case there is any calcification in a chest X-ray with right-sided heart failure symptoms, we should consider the diagnosis of constrictive pericarditis and performing further cardiac investigations.
